# The Persistent Gap in Mass Drug Administration Uptake Among Known Microfilaria-Positive Individuals in India's Lymphatic Filariasis Elimination Program: A Call for Strengthened Follow-Up Strategies

**DOI:** 10.7759/cureus.104607

**Published:** 2026-03-03

**Authors:** Ezhilarasan Selvaraju

**Affiliations:** 1 Community Medicine, Bhaarath Medical College and Hospital, Bharath Institute of Higher Education and Research (BIHER), Chennai, IND

**Keywords:** disease eradication, health policy, lymphatic filariasis, mass drug administration, microfilaria, neglected tropical diseases, program evaluation, public health surveillance

## Abstract

India’s lymphatic filariasis elimination program has achieved high mass drug administration (MDA) coverage and substantial reductions in transmission across endemic districts. As the program transitions toward transmission assessment surveys (TAS) and post-MDA surveillance, the strategic focus is shifting from expanding coverage to sustaining verified interruption of transmission. However, reliance solely on aggregate coverage metrics may overlook an important endgame vulnerability: incomplete longitudinal follow-up of individuals identified as microfilaria (Mf) positive or filarial test strip (FTS) positive during surveillance activities.

Although defined epidemiologic thresholds (microfilaremia <1% or antigenemia <2% in young children) guide decisions to stop MDA, achieving these benchmarks does not necessarily ensure that all previously identified infected individuals have cleared infection. In low-prevalence settings, even small numbers of untreated or inadequately treated individuals may sustain localized transmission. Strengthening case-based registries, treatment verification mechanisms, and structured follow-up of confirmed positives can enhance epidemiologic confidence in validated elimination and reduce the risk of recrudescence.

## Editorial

India’s lymphatic filariasis elimination program, implemented through annual mass drug administration (MDA), has substantially reduced transmission and narrowed endemicity across the country [1]. Many districts are transitioning from repeated treatment rounds to transmission assessment surveys (TAS) and post-MDA surveillance [2]. As elimination validation approaches, the program’s focus has shifted from expansion to consolidation, emphasizing sustained gains and prevention of resurgence.

Despite this progress, an important operational refinement deserves attention. While community-level MDA coverage often meets or exceeds recommended thresholds, structured longitudinal verification of treatment among individuals identified as microfilaria-positive or filarial test strip (FTS) positive may not be uniformly implemented across all settings. National guidelines mandate treatment of confirmed positives; however, during the transition from mass delivery to surveillance phases, case-based tracking systems may vary in strength across districts. Thus, the concern reflects both documented compliance gaps in field settings and a programmatic vulnerability during the elimination endgame.

Individuals who test positive during night blood surveys or are identified as FTS positive during pre-TAS and TAS represent the measurable human reservoir sustaining transmission [2]. As overall prevalence declines, the relative epidemiologic impact of each untreated infected individual increases. Even a small number of persistently untreated individuals can sustain low-level transmission and influence elimination outcomes.

Field observations from two districts of Jharkhand in 2023 August MDA round further illustrate this concern. Among 73 microfilaria-positive individuals identified through night blood surveys in sentinel and random sites, even after more than 5 rounds of MDA, 11 (15.1 percent) reported never having consumed MDA drugs in their lifetime despite multiple rounds. Of the 73 individuals, 47 (64.4 percent) were asymptomatic at the time of interview, while 22 (30.1 percent) had lower limb involvement. These findings suggest that reported coverage may not reliably reflect ingestion or sustained adherence among confirmed positives, particularly in low-prevalence settings.

Within the elimination framework, MDA discontinuation is guided by defined epidemiologic thresholds. Before stopping MDA in an implementation unit, infection levels must fall below critical cutoffs, typically microfilaremia below 1% or circulating filarial antigenemia below 2% in young children, to qualify for TAS [2]. These benchmarks appropriately guide the transition from mass treatment to surveillance. However, meeting district-level thresholds does not necessarily ensure that all previously identified infected individuals have cleared infection.

Although TAS are statistically robust for determining whether infection levels fall below predefined thresholds, they are cluster-based and not designed to verify clearance in every previously identified infected individual. Random sites are rotated across successive MDA rounds and surveillance cycles, and therefore, previously identified positive individuals may fall outside routine sampling frameworks. TAS, therefore, complement but do not replace individual-level follow-up mechanisms.

Individuals identified as microfilaria positive or FTS positive require continued annual antifilarial treatment and structured follow-up consistent with WHO guidance to ensure sustained parasite clearance and prevent recrudescence [2]. Current antifilarial regimens, including triple drug therapy, demonstrate high efficacy in clearing microfilaremia. However, therapeutic effectiveness depends on confirmed ingestion and annual adherence. Without systematic case-based tracking and documentation of treatment completion, residual reservoirs may persist even after population-level thresholds are achieved.

Evidence from Indian epidemiologic assessments further underscores this issue. In districts completing multiple rounds of MDA, microfilaremia has declined substantially, and antigenemia among children has fallen below TAS thresholds. Nevertheless, localized hotspots and spatial clustering have been identified [3], indicating that aggregate metrics may mask focal persistence.

Monitoring systems traditionally emphasize coverage indicators and post-MDA surveys. While essential for evaluating program reach, these approaches do not consistently confirm treatment completion among previously identified infected individuals. Studies from Indian settings demonstrate that coverage does not always translate into compliance, and gaps in drug ingestion remain a barrier to elimination [4].

Persistent non-uptake reflects both behavioral and structural determinants. Asymptomatic individuals may not perceive urgency for treatment. Fear of adverse reactions, misconceptions, migration, and community fatigue reduces compliance. Individual adherence is influenced by perceived risk, trust in health systems, and communication strategies [4]. Limited case-based tracking systems may weaken accountability across successive rounds.

Recent epidemiologic monitoring surveys further demonstrate that infection may persist in localized pockets even after multiple MDA rounds, reinforcing the need for strengthened surveillance and targeted follow-up [5]. Complementary vector surveillance and control measures may further strengthen post-MDA monitoring frameworks. Detection of infective larvae in Culex mosquitoes, along with estimation of vector infection and infectivity rates in identified hotspot areas, can provide early signals of residual transmission. WHO monitoring guidance recognizes entomological assessment and molecular Xeno monitoring as supportive tools in evaluating transmission interruption [2]. Integrating entomological indicators with human surveillance data may enhance confidence in sustained elimination and reduce the risk of recrudescence.

During high-burden phases, a coverage-driven strategy was appropriate and effective. In the elimination endgame, however, the focus must evolve from coverage percentage to confirmed reservoir clearance. Operational strengthening could include conducting targeted night blood surveys among previously identified microfilaria-positive or FTS-positive individuals, even if their villages are not selected as sentinel sites, random sites, pre-TSA sites, or TSA sites. Because random sites change across successive MDA rounds and surveillance cycles, previously identified positive individuals may fall outside routine sampling frameworks. Ensuring follow-up testing of such individuals can help document parasite clearance and reduce the risk of undetected residual reservoirs.

Additional strengthening measures could include district level digital registries of all microfilaria-positive and FTS-positive individuals linked to primary health centers to enable longitudinal follow-up; consideration of documented annual treatment completion for confirmed positives prior to TSA clearance; integration of infection specific follow up indicators into routine health management information systems; targeted hotspot mapping where clustering is detected; and strengthened community risk communication emphasizing the transmission role of asymptomatic infection.

These measures can be embedded within existing lymphatic filariasis elimination and primary healthcare platforms, minimizing additional resource burden while strengthening accountability.

India stands at a pivotal stage in its elimination journey. Achieving threshold coverage targets is necessary but not sufficient. Durable elimination depends on ensuring that no identified infection remains untreated. Embedding reservoir-focused accountability into routine program design will strengthen confidence in validated transmission interruption and secure long-term elimination success.
